# Dynamics of resistance mutations to NS3 protease inhibitors in a cohort of Brazilian patients chronically infected with hepatitis C virus (genotype 1) treated with pegylated interferon and ribavirin: a prospective longitudinal study

**DOI:** 10.1186/1743-422X-10-57

**Published:** 2013-02-14

**Authors:** Luísa Hoffmann, Juliene Antonio Ramos, Elizabeth Valentin de Souza, Ana Lucia de Araújo Ramos, Cristiane Alves Villela-Nogueira, Turán Péter Ürményi, Amilcar Tanuri, Edson Rondinelli, Rosane Silva

**Affiliations:** 1 Instituto de Biofísica Carlos Chagas Filho, Universidade Federal do Rio de Janeiro, Rio de Janeiro, Brazil; 2 Instituto Nacional para Pesquisa Translacional em Saúde e Ambiente na Região Amazônica, Conselho Nacional de Desenvolvimento Científico e Tecnológico/MCT, Rio de Janeiro, Brazil; 3 Instituto Federal de Educação, Ciência e Tecnologia do Rio de Janeiro, Rio de Janeiro, Brazil; 4 Departamento de Clínica Médica, Hospital Universitário Clementino Fraga Filho, Universidade Federal do Rio de Janeiro, Rio de Janeiro, Brazil; 5 Instituto de Biologia, Universidade Federal do Rio de Janeiro, Rio de Janeiro, Brazil

**Keywords:** HCV NS3 protease, Drug resistance persistency, Selection pressure, Antiviral drugs, Chronic Hepatitis C infection

## Abstract

**Abstract:**

About sixty thousand new cases of Hepatitis C virus (HCV) infection are recorded in Brazil each year. These cases are currently treated with pegylated interferon (PEG-IFN) and ribavirin (RBV) with an overall success rate of 50%. New compounds for anti-HCV therapy targeted to the HCV NS3 protease are being developed and some already form the components of licensed therapies. Mapping NS3 protease resistance mutations to protease inhibitors or anti-viral drug candidates is important to direct anti-HCV drug treatment.

**Methods:**

Sequence analysis of the HCV NS3 protease was conducted in a group of 68 chronically infected patients harboring the HCV genotype 1. The patients were sampled before, during and after a course of PEG-IFN-RBV treatment.

**Results:**

Resistance mutations to the protease inhibitors, Boceprevir and Telaprevir were identified in HCV isolated from three patients (4.4%); the viral sequences contained at least one of the following mutations: V36L, T54S and V55A. In one sustained virological responder, the T54S mutation appeared during the course of PEG-IFN and RBV therapy. In contrast, V36L and V55A mutations were identified in virus isolated from one relapsing patient before, during, and after treatment, whereas the T54S mutation was identified in virus isolated from one non-responding patient, before and during the treatment course.

**Conclusions:**

The incidence and persistence of protease resistance mutations occurring in HCV from chronically infected patients in Brazil should be considered when using protease inhibitors to treat HCV disease. In addition, patients treated with the current therapy (PEG-IFN and RBV) that are relapsing or are non-responders should be considered candidates for protease inhibitor therapy.

## Background

Infection with hepatitis C virus (HCV) is a global health problem. The World Health Organization (WHO) [1] estimates that 3% of world’s population is infected with HCV. Chronic HCV can lead to complications like cirrhosis and hepatocellular carcinoma (HCC), which are the main reasons for liver transplantation [2]. There are three million people infected with HCV in Brazil, with regional differences in the infection rates observed across the country [3]; these differences range from 0.28% in central and western regions to 0.62% in the north. According to the National Information System on Disease Notification (SINAN) of the Brazilian Ministry of Health, between 1999 and 2009, 60,908 new cases of hepatitis C were recorded [4].

Treatment of chronic hepatitis C infections with pegylated interferon (PEG-IFN) and ribavirin (RBV) results in a sustained virological response (SVR) in only about 50% of the patients treated. SVR is characterized by undetectable levels of HCV RNA when patients are assessed 24 weeks post-treatment. Lower response rates are found in individuals infected with the HCV genotype 1, the most common genotype worldwide [5]. In Brazil, some studies have described the prevalence of genotype 1 [3,6,7]. Hence, many direct-acting antiviral compounds are being considered for anti-HCV therapy [8]. The main targets are the viral protein RNA-dependent RNA polymerase (NS5B) [9]and the serine protease NS3/4A. Treatment success has been obtained with the NS3 protease inhibitors (PIs) Telaprevir [10,11] and Boceprevir [12,13], both of which have been approved for clinical use by the US Food and Drug Administration (FDA) and the Brazilian National Agency for Sanitary Vigilance (ANVISA). Studies on Serine Protease Inhibitor Therapy (SPRINT) have aimed to establish the safety and efficacy of boceprevir or telaprevir when added to standard peginterferon and ribavirin therapy; such studies have shown that sustained virological responses in patients can reach 70% [14-16]. However, resistance mutations against HCV PIs can limit the effectiveness of such treatments. Genetic variants that confer resistance to PIs are being found in treatment-naïve patients [17-23].

Because of the lack of a proofreading mechanism during HCV genome replication, extensive sequence variability in the viral population can be found in the sera of HCV-infected patients. The genetic variability of circulating HCVs can affect the treatment and persistence of the virus, and poses a great challenge for vaccine development [24]. Nevertheless, few studies have shown the presence of primary resistance mutations in the Brazilian population [25].

In this study, we analyzed the dynamics of the appearance of primary resistance mutations to NS3 protease inhibitors and identified specific mutations in the NS3 protease gene in a cohort of Brazilian patients chronically infected with HCV genotype 1. We investigated the presence of such resistance mutations in the cohort before, during, and after treatment with PEG-IFN and RBV.

## Results

### Genetic variability of the HCV isolates

Blood samples were taken from 81 patients before treatment (naïve) with PEG-IFN and RBV. However, 13 patients were removed from the study due to their loss at follow up. Viral RNA was extracted from the serum of the remaining 68 patients from which the NS3 protease gene region was amplified and sequenced. Of the 68 patients analyzed, 26 achieved a sustained virological response (SVR) (38.2%), 10 were relapsing (REL) (14.7%), while 32 were non-responders (NR) (47.1%). Table 1 summarizes the main characteristics of the study population. The 38 men and 30 women (median age 55) possessed body mass indices ranging from 25 to 27.

**Table 1:** Characteristics of the study groups

**Patient Characteristics**	**SVR (n = 26)**	**REL (n = 10)**	**NR (n = 32)**	**Total (n = 68)**
**Median Age**	54.00	56.00	56.00	55.00
**Median BMI**	25.96	27.15	24.58	25.49
**Male (%)**	62	40	56	56

REL, Relapsing; NR, Non-Responder; SVR, Sustained Virological Response; BMI, Body Mass Index.

No statistically significant differences were observed between the SVR, REL and NR groups (p > 0.4, G-test and Chi-square test).

The evolutionary parameters, dN and dS, along with dN/dS ratios were calculated to evaluate whether evolutionary selective pressures had acted on the predominant HCV sequences. The results of these tests showed negative selective pressure on the sequences as all of the dN/dS ratios were <1 (0.05 – 0.06). No statistically significant differences in genetic diversity were observed in the study groups using AMOVA and Fst analysis (data not shown).

### Monitoring NS3 protease resistance mutations over time

Despite the presence of NS3 amino acid mutations in viruses isolated from the 68 patients studied, 3 (4.4%) had well-known resistance mutations to NS3 protease inhibitors, namely, V36L, T54S and V55A mutations. The temporal dynamics of these mutations were monitored before, during and after treatment with PEG-IFN and RBV in the patients (Table 2). The T54S mutation was detected on the seventh day of treatment in one SVR patient. In contrast, the V36L and V55A mutations in one REL patient were detected before, during and after treatment. There was a period where HCV RNA became undetectable during treatment. Six months after the treatment ended, however, the mutations became detectable once more as a result of relapse responses. Otherwise, one NR patient had the NS3 T54S mutation at all of the time points until the treatment was interrupted. No amino acid substitutions in the catalytic triad H57, D81, and S139 of the NS3 protease (which leads to nonfunctional enzymes) were detected in any of the isolates studied (data not shown).

**Table 2:** Dynamics of well-known protease inhibitor resistance mutations in the viral NS3 protease gene over time from patients infected with the Hepatitis C virus

**Treatment**	**Time**	**Patient ID**		
		**(Genotype – Treatment response – Mutation)**		
**PEG-IFN/RBV**		**Pat A**	**Pat B**	**Pat C**
		**(1b – SVR – T54S)**	**(1a – REL – V36L/V55A)**	**(1a – NR –T54S)**
Before		T	36L/55A	S
During	48 h	T*	36L/55A	S
	7d	S**	36L/55A	S
	30d	***	***	S
	3 m	-	***	S
	6 m	-	-	S
	12 m	-	-	Nb
After	18 m	-	36L/55A	Nb

SVR, Sustained Virological Responder; REL, Relapsing; NR, Non-Responder, -, Undetectable HCV RNA; Nb, No blood was collected. h, hours; d, days; m, months.

Viral loads ranged from 4 × 10^4^ to 4 × 10^6^ unless indicated by *. Viral load: * 7.0 × 10^2^, ** 2.6 × 10^3^, and *** <500 IU/ml.

## Discussion

This study evaluated heterogeneity in the HCV NS3 protease sequences isolated from HCV from a group of Brazilian patients with chronic hepatitis C whose responses to PEG-IFN and RBV treatment (SVR, REL and NR) were being monitored. We assessed genetic variability and the presence of protease inhibitor resistance mutations in the NS3 protease in the isolates before, during, and after treatment in patients with viral loads > 500 IU/ml. We investigated specific protease inhibitor resistance mutations, namely, V36A/L/M/G, Q41R, F43C/S, T54S/A, V55A, Q80R/K, S138T, R155K/T/G/I/Q/M/S/L and A156S/T/V/I. Of the 68 patients analyzed 3 (4.4%), had at least one of the mutations known to confer telaprevir and boceprevir resistance (V36L, T54S and V55A). These resistance mutations have been described previously [17-22]. In Brazil, knowledge is lacking about the temporal changes that occur in PI resistance mutations during the course of IFN/RBV treatment. One report [25] described the presence of V36L and T54S in HCVs in a group of Brazilian patients chronically infected with HCV. Recent studies have also highlighted the presence of naturally ocurring mutations in the NS3 protease in patients. For example, a study of four Spanish patients identified V36A, T54A, V55A, R155S, and A156T/V mutations in the NS3 protease [26]. In addition, a Japanese study identified T54S, Q80K, I153V and D168E NS3 protease mutations among 261 patients (13.4% had a single mutation, while 2.3% had double mutations) [27]. NS3 protease mutations (V36L, T54S, V55A/I, and Q80K/L) were also observed in 29% of genotype 1a patients in an Italian population [28].

In the present study, V36L, T54S and V55A mutations were identified in Brazilian patients treated with PEG-IFN and RBV. To assess the maintenance of protease mutation variants, this analysis was performed before, during and after treatment completion, thus allowing us to determine the fate of the genetic variants over an 18 months period. Negative selective pressure was observed acting on the NS3 protease region analyzed here. In addition, the observed mutations may confer resistance to telaprevir and boceprevir, making it possible to predict that patients harboring viruses with such mutations may not benefit from a future treatment with these drugs; therefore, the null responder patients should be engaged in the new Protease Therapy Protocol [10,12,16,29]. These two drugs (Boceprevir and Telaprevir), already approved by FDA and by ANVISA in Brazil [30], are now components of licensed therapies. Thus, mapping NS3 protease resistance mutations to protease inhibitors may be an important tool to direct anti-HCV treatment.

## Methods

### Study population

Patients were recruited between April 2006 and August 2008 at the Hepatology Clinic of the Hospital Universitário Clementino Fraga Filho (HUCFF), Rio de Janeiro (RJ), Brazil. Patients chronically infected with HCV showing liver disease and detectable HCV RNA were treated with PEG-interferon α-2b (1.5 μg/kg once a week) and ribavirin (1,000-1,250 mg/day according to body weight and patients weighing more than 75 kg received 1,250 mg/day) for 12 months. Patients with undetectable serum HCV RNA 6 months after treatment were considered to have sustained virological responses (SVR). REL are patients that have undetectable viral loads (serum) during treatment and relapses after 6 months of treatment. Patients who fail to suppress serum HCV RNA by at least 2 logs after three months of treatment were considered to be non-responders (NR). Blood samples were collected from 68 patients infected with the HCV genotype 1a (n = 37) or 1b (n = 31) before (treatment-naïve), during (48 hours, 7 days, 30 days, 3 months, 6 months, 12 months) and 6 months after the end of treatment to evaluate SVR.

### Ethical approval

Written informed consent was obtained from every patient, previously approved by the ethics committee of the Hospital of Federal University of Rio de Janeiro by the number 166/05 in 2005.

### Viral RNA isolation

HCV RNA was isolated from 100μL of serum using a QIAamp® MinElute® Virus Spin kit (Qiagen Inc., Valencia, CA). Isolation was performed according to manufacturer’s instructions.

### RT-PCR amplification of the NS3 protease gene

Reverse transcription (RT) was done using the High Capacity cDNA Archive Kit (Applied Biosystems, Inc., Foster City, California, USA) according to manufacturer's instructions. A nested PCR was done to amplify the NS3 protease gene using degenerate external forward (5^′^ GCDCCYATYACRGCBTAYKCYCARCAGAC 3^′^) and reverse (5^′^ ACYTTRGTGCTYTTRCCGCTRCCRGTGGG 3^′^) primers and internal forward (5^′^ CBTAYKCYCARCAGACRMGRGGC 3^′^) and reverse (5^′^ GGRGWBGARTTRTCYGWRAABACCGG 3^′^) primers. The PCR mixture for both rounds of nested PCR contained 0.15 mM dNTPs, 2 mM MgCl_2_, 0.3 mM forward and reverse primers and 1 unit of Platinum *Taq* DNA polymerase (Invitrogen, Carlsbad, CA, USA). The PCR protocol consisted of 5 min at 94°C, 30 cycles of 30 s at 94°C, 30 s at 55°C and 60 s at 72°C, and a final 10 min at 72°C. The resulting 555-bp PCR products were purified using the Montage PCR Centrifugal Filter Devices (Millipore, Bedford, MA, USA) before sequencing.

### Sequence analyses of the viral NS3 protease gene

Purified PCR products were directly sequenced using internal forward and reverse primers. Amplicons were sequenced using a Big Dye® Terminator v3.1 Cycle Sequencing Kit (Applied Biosystems, Inc., Foster City, California, USA) and ABI Genetic Analyzer 3130. Sequence analysis was done using Geneious 4.7.5 [31] and MEGA 4.1 software. All of the sequences obtained were submitted to GenBank under the following consecutive accession numbers: JX106306 to JX106345. Amino acid sequences were aligned and compared to the HCV reference sequence (1a genotype H77) (accession number AF009606) [32,33]. Evidence of evolutionary selective pressure on several aligned predominant sequences was determined by calculating the dN/dS ratio, where dN represents a non-synonymous site in which the nucleotide alteration leads to an amino acid change, while dS is a synonymous site in which the nucleotide alteration does not change the corresponding amino acid. dN/dS ratios were calculated using the Synonymous Non-Synonymous Analysis Program (SNAP) [34]. Genetic diversity (d) between the most common sequences was assessed using MEGA 4.1 software with the nucleotide model Maximum Composite Likelihood. An Analysis of Molecular Variance (AMOVA) and population pairwise Fst vales were assessed using Arlequin software with the Tamura 3 parameter as the distance method.

## Abbreviations

ANVISA: National Agency *for* Sanitary Vigilance; BMI: Body mass index; DAAs: Direct antiviral agents; FDA: Food and Drug Administration; HCC: Hepatocellular carcinoma; HCV: Hepatitis C virus; IFN: Interferon; ORF: Open reading frame; PEG: Pegylated; PIs: Protease inhibitors; RBV: Ribavirin; REL: Relapsing; SINAN: National Information System on Disease Notification; SVR: Sustained virological responder; WHO: World Health Organization.

## Competing interest

The authors declare that they have no competing interests.

## Authors’ contributions

LH contributed to the study design, data acquisition and analysis and drafted the manuscript; JAR was involved in data acquisition and revision of the manuscript; EVS, was involved in data acquisition and analysis; ALAR worked on aspects of the study relating to the cohort of patients with chronic hepatitis C; CAVN worked on aspects of the study relating to the cohort of patients with chronic hepatitis C; TPU was involved in data acquisition and revision of the manuscript; AT: contributed to the study design and drafted the manuscript; ER contributed to the study design, data-analysis, and drafted the manuscript. RS: contributed to the study design, data acquisition and analysis, and revised the manuscript. All authors have read and approved the final manuscript.
